# Assessment of early metabolic progression in melanoma patients under immunotherapy: an ^18^F-FDG PET/CT study

**DOI:** 10.1186/s13550-021-00832-4

**Published:** 2021-09-08

**Authors:** Christos Sachpekidis, Annette Kopp-Schneider, Jessica C. Hassel, Antonia Dimitrakopoulou-Strauss

**Affiliations:** 1 Clinical Cooperation Unit Nuclear Medicine, German Cancer Research Center (DKFZ), Im Neuenheimer Feld 280, 69210 Heidelberg, Germany; 2 Department of Biostatistics, German Cancer Research Center (DKFZ), Heidelberg, Germany; 3 Department of Dermatology and National Center for Tumor Diseases (NCT), University Hospital Heidelberg, Heidelberg, Germany

**Keywords:** Metastatic melanoma, Immunotherapy, ^18^F-FDG PET/CT, Unconfirmed progressive disease, Pseudoprogression, Confirmed progressive disease, Spleen glucose metabolism

## Abstract

**Background:**

The usage of immune checkpoint inhibitors (ICIs) is the standard practice for the treatment of metastatic melanoma. However, a significant amount of patients show no response to immunotherapy, while issues on its reliable response interpretation exist. Aim of this study was to investigate the phenomenon of early disease progression in 2-deoxy-2-(^18^F)fluoro-D-glucose (^18^F-FDG) positron emission tomography/computed tomography (PET/CT) in melanoma patients treated with ICIs.

**Methods:**

Thirty-one patients under ICIs serially monitored with ^18^F-FDG PET/CT were enrolled. All patients exhibited progressive metabolic disease (PMD) after two ICIs’ cycles according to the European Organization for Research and Treatment of Cancer (EORTC) criteria, and were characterized as unconfirmed PMD (uPMD). They were further followed with at least one PET/CT for either confirmation of PMD (cPMD) or demonstration of pseudoprogression remission. Patients were also evaluated with the PET Response Evaluation Criteria for Immunotherapy (PERCIMT). Moreover, in an attempt to investigate immune activation, the spleen to liver ratios (SLR_mean_, SLR_max_) of ^18^F-FDG uptake were measured.

**Results:**

Median follow up was 69.7 months [64.6–NA]. According to EORTC, 26/31 patients with uPMD eventually showed cPMD (83.9%) and 5/31 patients showed pseudoprogression (16.1%). Patients with cPMD (*n* = 26) had a median OS of 10.9 months [8.5–NA], while those with pseudoprogression (*n* = 5) did not reach a median OS [40.9–NA]. Respectively, after application of PERCIMT, 2/5 patients of the pseudoprogression group were correctly classified as non-PMD, reducing the uPMD cohort to 29 patients; eventually, 26/29 patients demonstrated cPMD (89.7%) and 3/29 pseudoprogression (10.3%). One further patient with pseudoprogression exhibited transient, sarcoid-like, mediastinal/hilar lymphadenopathy, a known immune-related adverse event (irAE). Finally, patients eventually showing cPMD exhibited a significantly higher SLR_mean_ than those showing pseudoprogression after two ICIs’ cycles (*p* = 0.038).

**Conclusion:**

PET/CT, performed already after administration of two ICIs’ cycles, can identify the majority of non-responders in melanoma immunotherapy. In order to tackle however, the non-negligible phenomenon of pseudoprogression, another follow-up PET/CT, the usage of novel response criteria and vigilance over emergence of radiological irAEs are recommended. Moreover, the investigation of spleen glucose metabolism may offer further prognostic information in melanoma patients under ICIs.

## Background

In recent years the clinical application of novel immune checkpoint inhibitors (ICIs) has constituted a major breakthrough in the management of advanced melanoma, leading to unprecedented response and survival rates [1, 2]. ICIs are monoclonal antibodies that promote tumoricidal effects by targeting regulatory pathways in T-cells. The two most effective classes of ICIs are directed towards the cytotoxic T-lymphocyte-associated protein 4 (CTLA-4; ipilimumab) or the programmed cell death protein 1 (PD-1; nivolumab, pembrolizumab) [3, 4]. The usage of ICIs is considered nowadays the standard practice for the treatment of metastatic melanoma [5]. Despite these improvements, however, a significant amount of patients—approximately 40–45%—show no response to immunotherapy [6].

Immunotherapeutic agents act markedly different than usual cytotoxic approaches, notably by generating inflammations rather than direct lysis. This unique mechanism of action can lead to novel response patterns, which pose relevant challenges in the interpretation of treatment response by conventional imaging approaches [7]. Pseudoprogression, defined as an initial increase in tumor burden followed by tumor regression, represents a distinct, atypical response pattern, initially described in melanoma patients undergoing ipilimumab therapy [8, 9]. Pseudoprogression is considered the result of a transient immune cell infiltration of the tumor [9, 10]. Another biologic explanation for the phenomenon could be a continued tumor cell growth until a sufficient response to immunotherapy takes place [11]. Regardless of etiology, since pseudoprogression may be misclassified as progressive disease, its reliable and early identification would offer significant therapeutic implications in patient management.

In order to capture the atypical patterns of tumor response described with ICIs, several modified radiologic response criteria have been proposed, including the immune-related response criteria (irRC) [9], the immune-related response evaluation criteria in solid tumors (irRECIST) [12], the immune response evaluation criteria in solid tumors (iRECIST) [13], and the immune-modified response evaluation criteria in solid tumors (imRECIST) [14]. Despite their differences, all these criteria require or at least recommend a confirmation of progressive disease in follow-up CT or MRI scans [15]. Similar attempts have been made with 2-deoxy-2-(^18^F)fluoro-D-glucose (^18^F-FDG) positron emission tomography/computed tomography (PET/CT), leading to the proposal of the respective, metabolic criteria, namely the PET/CT Criteria for Early Prediction of Response to Immune Checkpoint Inhibitor Therapy (PECRIT) [16], the PET Response Evaluation Criteria for Immunotherapy (PERCIMT) [17], the immune PET Response Criteria in Solid Tumors (iPERCIST) [18] and the immunotherapy-modified PET Response Criteria in Solid Tumors (imPERCIST) [19]. However, the PET-based approaches have included smaller patient cohorts than the radiologic ones.

Considering the potential benefit of reliably identifying the non-negligible number of non-responders early during immunotherapy, we aimed to investigate the phenomenon of early metabolic disease progression in ^18^F-FDG PET/CT in metastatic melanoma patients under ICIs.

## Methods

### Patients

Thirty-one metastatic melanoma patients under ICIs were enrolled in this retrospective analysis (23 males, 8 females; mean age 56.2 years) of a prospective study based on the criteria described in the next paragraph. Some of these patients have been analyzed in other publications but with different approaches than in the here presented study [20, 21]. All patients underwent immunotherapy with a CTLA-4 inhibitor (ipilimumab; *n* = 26 patients), a PD-1 inhibitor (pembrolizumab; *n* = 1 patient) or a combination ICI treatment of a CTLA-4 and a PD-1 inhibitor (ipilimumab/nivolumab; *n* = 4 patients). Ipilimumab was administered intravenously at a dose of 3 mg/kg every 3 weeks for a total of 4 doses. Pembrolizumab was administered intravenously at a dose of 2 mg/kg every 3 weeks. The combination ICIs therapy was administered as an induction of 4 cycles of nivolumab (1 mg/kg) and ipilimumab (3 mg/kg) every 3 weeks, followed by single-agent nivolumab administration (3 mg/kg) every 2 weeks.

For the reasons of this study, patient inclusion criteria involved: (a) PET/CT imaging of each patient at least 3 times, definitely including the following time-points: baseline before the start of immunotherapy, early during therapy (after two cycles), and after completion of four cycles of ICIs’ treatment. (b) Demonstration of initial/early signs of disease progression on PET/CT after 2 cycles of therapy according to the European Organization for Research and Treatment of Cancer (EORTC) criteria for PET [22]. In terms of the present analysis, these patients were characterized as demonstrating unconfirmed progressive metabolic disease (uPMD). (c) No intermediate changes in systemic treatment after initial exhibition of signs of progression on PET/CT (uPMD) and until definition of clinical disease progression, as determined by the dermato-oncologists (JCH). Clinical progression was based on a combination of clinical follow-up, standard of care imaging—including CT, PET/CT and brain MRI—as well as serum levels of the tumor marker lactate dehydrogenase (LDH).

Patients gave written informed consent to participate in the study and to have their medical records released. The study was approved by the Ethical Committee of the University of Heidelberg (S-107/2012) and the Federal Agency for Radiation Protection (Bundesamt für Strahlenschutz, Z 5-22463/2-2012-016).

### ^18^F-FDG PET/CT data acquisition

All patients underwent serial, whole body PET/CT imaging acquired 60 min after intravenous administration of maximum 250 MBq ^18^F-FDG. PET/CT was performed from the head to the feet with an image duration of 2 min per bed position. A dedicated PET/CT system (Biograph mCT, S128, Siemens Co., Erlangen, Germany) with an axial field of view of 21.6 cm with TruePoint and TrueV, operated in a three-dimensional mode was used. A low-dose attenuation CT (120 kV, 30 mA) was used for attenuation correction of the PET data and for image fusion. All PET images were attenuation-corrected and an image matrix of 400 × 400 pixels was used for iterative image reconstruction. Iterative images reconstruction was based on the ordered subset expectation maximization (OSEM) algorithm with point spread function modelling (2 iterations and 21 subsets) as well as time of flight.

### ^18^F-FDG PET/CT data analysis

PET/CT images were analyzed on an Aycan workstation by two nuclear medicine physicians (CS, ADS) and interpreted by consensus. Visual analysis was based on the identification of sites of focal, non-physiologic ^18^F-FDG uptake above surrounding background activity, considered consistent with melanoma lesions. All ^18^F-FDG-avid lesions were correlated with the fused low-dose CT findings in order to enhance the diagnostic certainty. Moreover, patterns of ^18^F-FDG uptake suggestive of radiologic manifestations of immune-related adverse events (irAEs) to immunotherapy were recognized and discerned from tumor manifestations. In particular, a new, diffusely enhanced tracer uptake in organs such as the gastrointestinal tract (mostly elongated uptake in the colon), the thyroid gland and in the bone marrow, or respectively, a new, relatively symmetrical, increased uptake in joints following ICIs were considered consistent with radiologic irAEs and not metastases.

Metabolic response to treatment was based on the EORTC criteria and the PERCIMT (Table 1) [17, 22]. As mentioned above, only patients with early disease progression (after 2 ICIs’ cycles) according to EORTC (characterized as uPMD) were enrolled in the analysis. All patients were followed with at least one additional PET/CT scan, performed after an interval of a minimum of six weeks, i.e. after administration of the first four cycles of treatment. According to the findings of the latest follow-up scan, without any intermediate changes in treatment, two categories of response were established: (a) confirmed progressive metabolic disease (cPMD), defined as persisting PMD with no signs of remission, and (b) pseudoprogression, defined as metabolic response (partial metabolic response, PMR, or complete metabolic response, CMR) at some time-point after the initial increase in total tumor burden.

**Table 1:** Summary of the EORTC and PERCIMT response criteria

	EORTC	PERCIMT
CMR	Complete resolution of ^18^F-FDG uptake within the tumor volume	Complete resolution of all pre-existing^18^F-FDG avid lesions. No new, ^18^F-FDG avid lesions
PMR	Decrease in tumor SUV > 25% after more than 1 therapeutic cycle	Complete resolution of some pre-existing^18^F-FDG avid lesions. No new, ^18^F-FDG avid lesions
SMD	Increase in tumor SUV < 25% or decrease in SUV < 15%	Neither PMD nor PMR/CMR
PMD	Increase in tumor SUV > 25% or appearance of new lesions	≥ 4 new lesions of less than 1.0 cm in functional diameter or ≥ 3 new lesions between 1.0 and 1.5 cm in functional diameter or ≥ 2 new lesions of more than 1.5 cm in functional diameter

EORTC, European Organization for Research and Treatment of Cancer; PERCIMT, PET Response Evaluation Criteria for Immunotherapy; CMR, complete metabolic response; PMR, partial metabolic response; SMD, stable metabolic disease; PMD, progressive metabolic disease; SUV, standardized uptake value

Further, the standardized uptake values (SUV_mean_, SUV_max_) of the liver and the spleen, if without disseminated metastatic disease, were measured in the PET/CT scans performed before treatment and after the first 2 cycles of ICIs. SUV values of the spleen were calculated from a central volume of interest (VOI) on the spleen, while the respective values of the liver were calculated from a VOI placed on the right liver lobe [23]. VOIs were drawn using the pseudo-snake algorithm of the Pmod software [24]. Based on these measurements, the spleen to liver SUV ratios (SLR_mean_, SLR_max_) were calculated separately for the groups of patients eventually showing cPMD and pseudoprogression.

### Statistical analysis

Statistical analysis included descriptive statistics, Welch's t-test for comparison of the SLR values between the two different patient groups as well as survival analysis. Median follow-up time was determined from start of treatment by inverse Kaplan–Meier estimation. Since time to confirmation of cPMD/pseudoprogression was different for cPMD and pseudoprogression, a landmark analysis was performed to compare overall survival (OS) between the two groups. OS was hence measured from the landmark, the date of the PET/CT with signs of cPMD or remission of pseudoprogression, until death from any cause or last follow-up. Kaplan–Meier estimates were generated and median OS [95% confidence interval] estimated. For univariate comparison of OS, a log-rank test was used. Statistical analysis was performed using R version 4.0.2 (The R Foundation for Statistical Computing 2020) and R packages survival and prodlim. The results were considered significant for *p* values less than 0.05 (*p* < 0.05).

## Results

### PET/CT findings

Each patient underwent a median number of 3 PET/CT scans (mean = 4.5 scans; range = 3–14 scans) between start of treatment and clinical disease progression. According to EORTC, 26 patients of the initial uPMD cohort (*n* = 31 patients) eventually demonstrated cPMD (83.9%), while 5 of them showed pseudoprogression (16.1%). Concerning the pseudoprogression group, 4/5 patients were under ipilimumab, while 1/5 patient was under combination treatment of ipilimumab/nivolumab. The median time between the emergence of signs of uPMD and confirmation of PMD was 1.5 months [1.4–1.9 months], while the median time between uPMD and remission of pseudoprogression was 2.3 months [1.5–NA months] (*p* = 0.092). The demographic characteristics, imaging findings and survival data of the five patients with pseudoprogression according to EORTC are summarized in Table 2.

**Table 2:** Characteristics of the patients with pseudoprogression after two cycles of ICIs according to EORTC

Patient number	Age (gender)	Therapy	^18^F-FDG PET/CT findings	Interval between pseudoprogression and remission (months)	Death	OS (months)	Method for potential identification of pseudoprogression
1	50 (M)	Ipilimumab/ nivolumab	Generalized lymphadenopathy	2.3	Yes	26.9	–
2	55 (M)	Ipilimumab	Two newly emerging cervical lesions (one cutaneous and one subcutaneous)	1.4	No	84.4	Application of PERCIMT
3	67 (M)	Ipilimumab	Increase in ^18^F-FDG activity and volume in existing tumor lesions	6.0	No	66.7	Application of PERCIMT
4	61 (F)	Ipilimumab	Sarcoid-like lymphadenopathy, pneumonitis, thyroiditis	1.4	Yes	43.5	Identification of irAEs
5	61 (M)	Ipilimumab	Increase in ^18^F-FDG activity, volume and number of tumor lesions	2.3	No	96.1	–

F, female; M, male; ICIs, immune checkpoint inhibitors; OS, overall survival; PERCIMT, PET Response Evaluation Criteria for Immunotherapy; irAEs, immune-related adverse-events

**Fig. 1:**
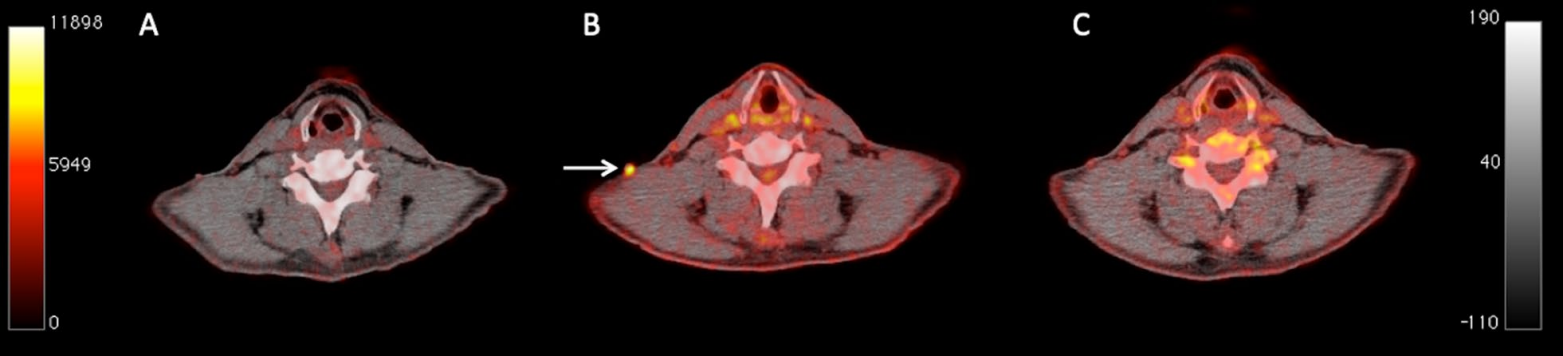
Transaxial PET/CT images at the cervical level of a 55-year-old male melanoma patient undergoing immunotherapy with ipilimumab (**a**–**c**). The PET/CT images before immunotherapy showed no pathologic lesions (**a**). Interim PET/CT performed after two cycles of ipilimumab demonstrated a new, small, ^18^F-FDG-avid subcutaneous lesion in the patient’s neck (white arrow; **b**), suspicious of metastatic involvement (uPMD, according to EORTC). A third PET/CT obtained soon after administration of four cycles of ipilimumab showed remission of the lesion (**c**), suggesting pseudoprogression of the cervical finding on interim PET/CT. The application of PERCIMT classified the patient as SMD already at the time of interim PET/CT, thus avoiding misinterpretation. At last follow-up the patient was still alive having reached an OS of 84.4 months

**Fig. 2:**
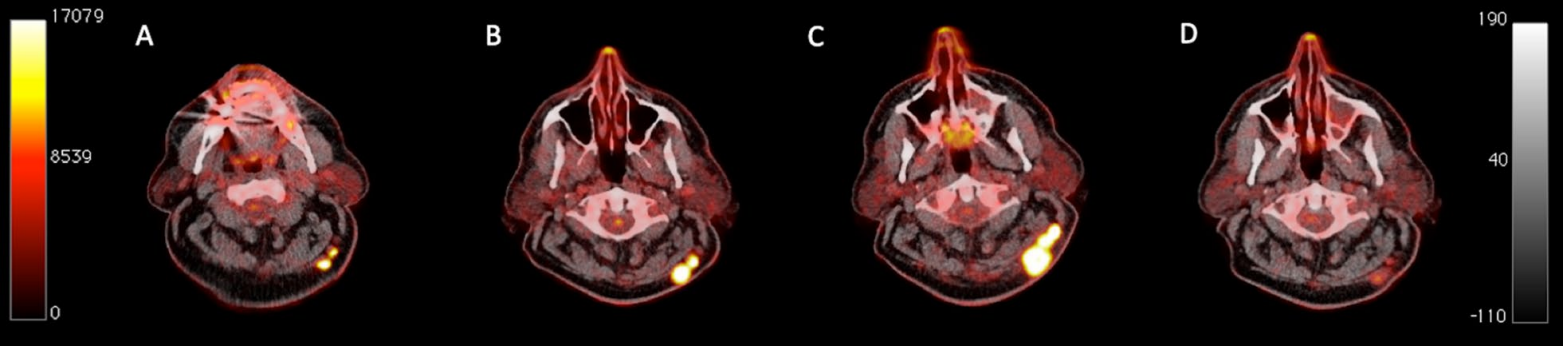
Transaxial PET/CT images at the cervical level of a 67-year-old melanoma patient undergoing immunotherapy with ipilimumab (**a**–**d**). Baseline PET/CT before commencement of therapy showed two hypermetabolic, subcutaneous metastases in the patient’s neck with SUV_max_ reaching 21.2 (**a**). After two cycles of ipilimumab, an increase in size and metabolism of the metastases was observed (SUV_max_ 28.8), leading to uPMD according to EORTC (**b**). The patient received two more cycles of ipilimumab and was re-examined with PET/CT, which demonstrated persistence of the hypermetabolic disease (SUV_max_ 25.5) and a further increase in lesions’ size (**c**). No further treatment was administered and, 4 months later, PET/CT demonstrated a marked morphologic and metabolic (SUV_max_ 4.2) remission of the lesions (**d**). Similar to the patient in Fig. 2, the application of PERCIMT classified the patient as SMD, thus tackling the phenomenon of pseudoprogression. At last follow-up the patient was still alive having reached an OS of 66.7 months

Compared to EORTC, the application of PERCIMT classified 2/5 patients of the above-mentioned pseudoprogression group as stable metabolic disease (SMD), since they did not fulfill the requirements for progression according to these criteria. In particular, one patient developed two small, cervical, ^18^F-FDG-avid lesions (Fig. 1), while the second one showed an increase in SUV and volume of the baseline tumor lesions without, however, developing new lesions (Fig. 2). Hence, according to PERCIMT, the total number of patients with uPMD was reduced to 29. Eventually, 26/29 patients demonstrated cPMD (89.7%), and 3/29 pseudoprogression (10.3%). The median time between uPMD and cPMD was 1.5 months [1.4–1.9 months], compared to 2.3 months [1.5–NA months] between uPMD and remission of pseudoprogression (*p* = 0.4).

With further consideration to the pseudoprogression group, one of the remaining three patients developed a transient, almost symmetric, sarcoid-like involvement of the mediastinal and hilar nodes, which is recognized as a possible, special type of irAE [25], accompanied by PET signs of pneumonitis and thyroiditis. Finally, the last two patients did not demonstrate a distinct pattern, potentially recognizable as pseudoprogression: the former showed a generalized lymphadenopathy with several newly emerging nodes, which reversed in the next scan. The latter exhibited a marked advance in tumor metabolic burden under treatment with both an increase in metabolism of the baseline lesions as well as development of several new lesions, which subsided after 6 months.

### Spleen to liver ratio (SLR) measurements

After performing semi-quantitative (SUV) measurements in the spleen and liver, the following SLR results at baseline (before commencement of immunotherapy) imaging were revealed: the mean SLR_mean_ of patients eventually showing cPMD was 0.91 (median = 0.92), while the mean SLR_mean_ of those eventually showing pseudoprogression was 0.85 (median = 0.85) (*p* = 0.062). Respectively, the baseline mean SLR_max_ of patients eventually showing cPMD was 0.85 (median = 0.87), compared to a mean SLR_max_ of 0.89 (median = 0.85) for patients with pseudoprogression (*p* = 0.625).

With regard to the measurements derived from PET/CT after the first 2 cycles of ICIs, patients eventually showing cPMD had a mean SLR_mean_ of 0.96 (median = 0.97), while patients with pseudoprogression had a mean SLR_mean_ of 0.88 (median = 0.88) (*p* = 0.038). Finally, the mean SLR_max_ values for patients with cPMD and pseudoprogression were 0.95 (median = 0.92) and 0.84 (median = 0.80), respectively (*p* = 0.112).

### Survival analysis

**Fig. 3:**
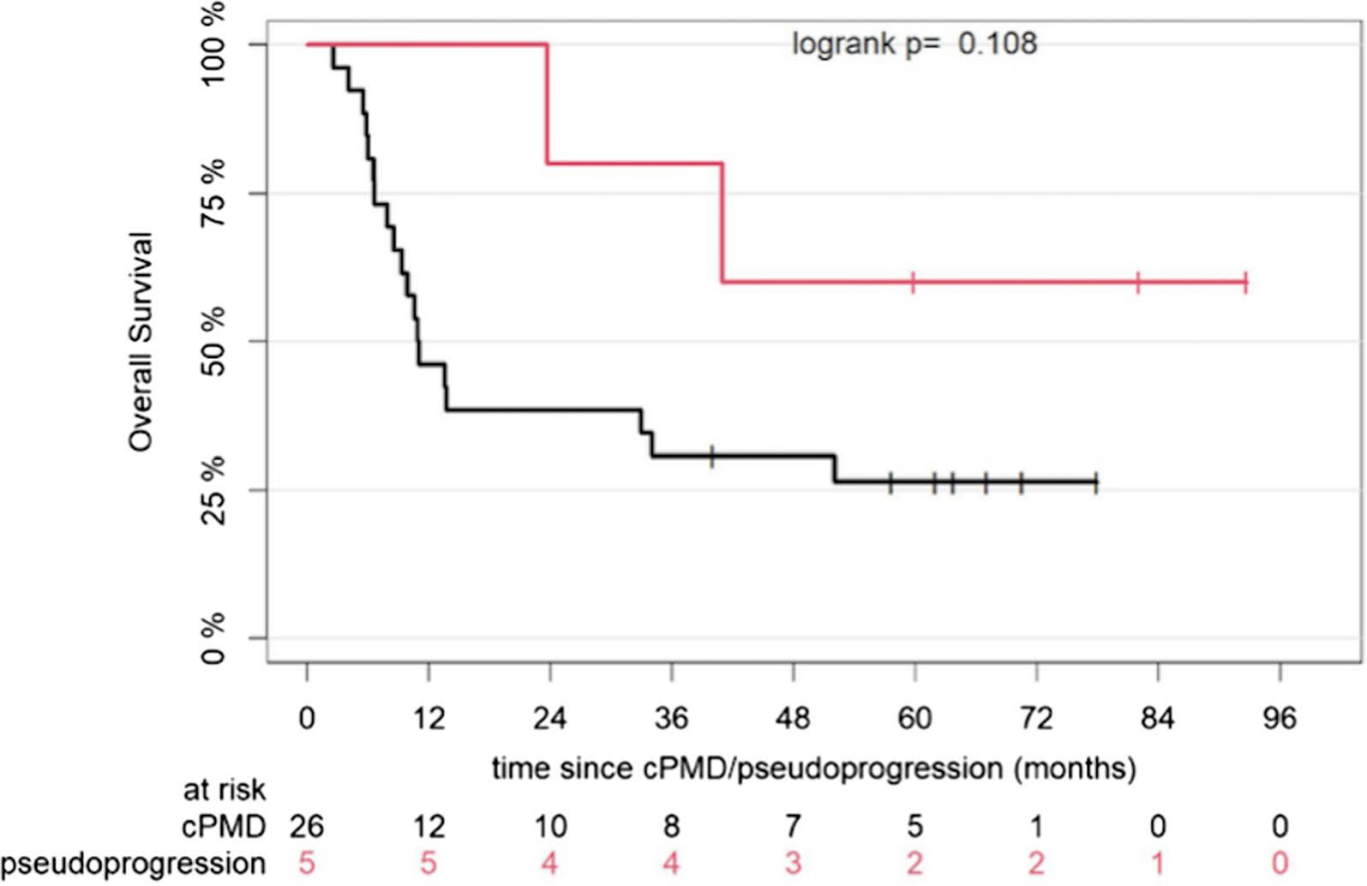
Kaplan–Meier estimates of OS according to response pattern after application of EORTC. The numbers of patients at risk in each group and for the respective time-points are shown below the plots. cPMD, confirmed progressive metabolic disease

**Fig. 4:**
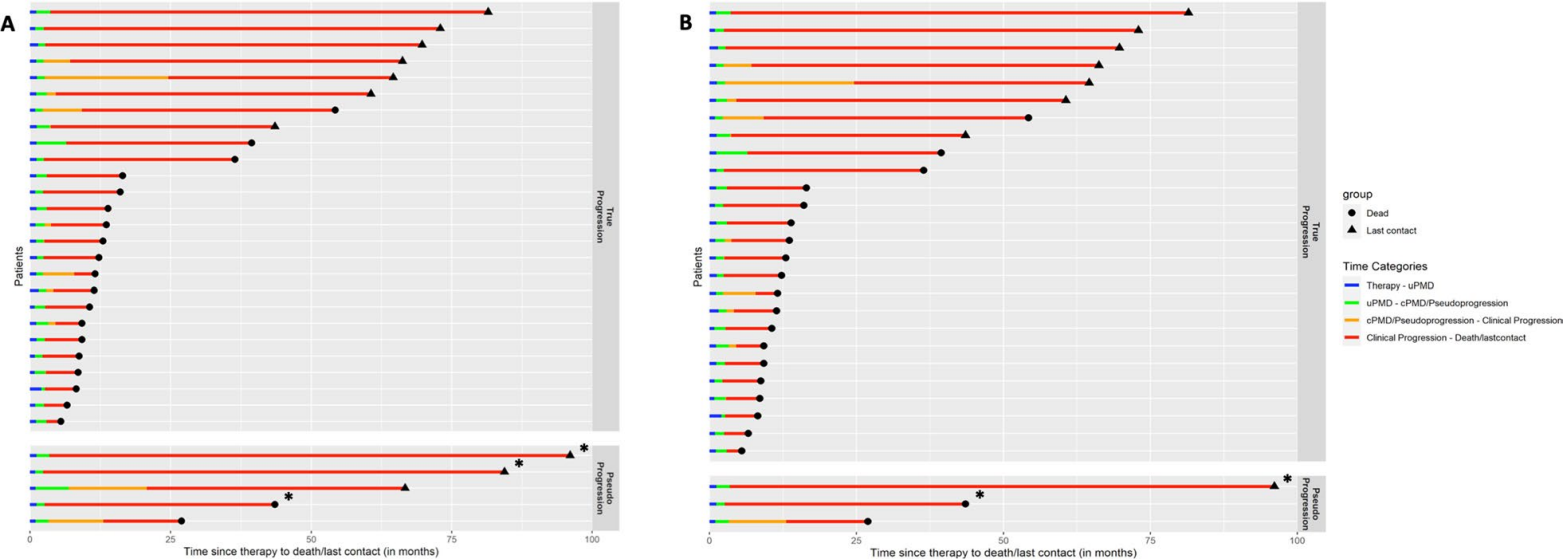
Plots of events of the studied patient cohort during the follow-up period after application of EORTC (*n* = 31 patients) (**a**) and PERCIMT (*n* = 29 patients) (**b**). The events depicted are: date of therapy commencement, date of uPMD, date of cPMD or remission of pseudoprogression, date of clinical progression, and date of death or last contact. For both sets of criteria, patients were dichotomized in those showing true progression/cPMD (upper part of the figures), and those showing pseudoprogression (lower part of the figures). *These patients did not show clinical disease progression according to the evaluation of the dermato-oncologists. In these cases, the red part of the lines of events corresponds to the time interval between remission of pseudoprogression in PET/CT and date of death or last contact

Median follow up of the patient cohort from start of treatment was 69.7 months [64.6–NA]. According to EORTC, patients showing cPMD (*n* = 26) had a median OS of 10.9 months [8.5–NA], while those with pseudoprogression (*n* = 5) did not reach a median OS [40.9–NA] (*p* = 0.108) (Fig. 3). Due to the small number of patients in the pseudoprogression group (*n* = 3), we refrained from performing the respective OS comparison based on PERCIMT. Figure 4 demonstrates the plots of the most significant events (trajectories) of the studied patient cohort during the follow-up period after application of both sets of criteria. These events include the date of ICIs’ therapy commencement, the date of uPMD on PET/CT, the date of cPMD or remission of pseudoprogression on follow-up PET/CT, the date of clinical progression—as defined by the dermato-oncologists—leading to cessation or change of the applied systemic treatment, as well as the date of death or last contact with the patients.

## Discussion

Through the restoration of T cell function, ICIs activate the adaptive immune system to produce enhanced anti-tumor responses [26]. The activation, differentiation and functions of T-cells are regulated by glucose metabolism. In particular, the “Warburg effect”, originally used to describe the shift of cancer cells from an aerobic mitochondrial oxidative metabolism to aerobic glycolysis to cover their demands, is also a key process for the sustainability of activated lymphocyte metabolism [27–29]. At the same time, the Warburg effect constitutes the fundamental of molecular imaging with ^18^F-FDG PET/CT in oncology [30]. In this context, the aforementioned similarity of cancer cell and activated lymphocyte metabolism—perfectly suited to match their functional needs [31]—leads to manifestation of both cell types by means of ^18^F-FDG PET/CT, inevitably, raising the issue of specificity.

The reliable differentiation of true disease progression from pseudoprogression is clinically relevant, since it can identify non-responders who will have a shorter duration of benefit from the treatment [32, 33]. However, the early and valid stratification of response to ICIs remains yet an insufficiently addressed issue [26, 34, 35]. We have previously highlighted the contribution of PET/CT in the reliable differentiation of metabolic responders from non-responders early during immunotherapy, stressing, however, the emergence of signs of pseudoprogression, which should be taken into consideration during PET/CT interpretation [20]. In the present study we tried to address this diagnostic challenge, focusing our investigation exclusively on melanoma patients exhibiting early signs of progression (uPMD) on ^18^F-FDG PET/CT under ICIs. Following the recommendations of the novel response criteria to immunotherapy [13, 18], we studied these patients with at least one additional PET/CT after the demonstration of uPMD in order to confirm or exclude the diagnosis of disease progression. A strength of the study is that serial PET/CT monitoring included at least 3 strictly defined and clinically relevant time-points during the course of immunotherapy: (a) shortly before the start of treatment, (b) early during therapy (after the 2 first cycles), and (c) soon after administration of 4 cycles of ICIs.

There are three major findings from our analysis. Firstly, the majority (83.9%) of patients with signs of early metabolic progression on PET/CT—already after administration of two ICIs cycles—eventually show confirmed progressive disease. On the other hand, approximately every sixth patient with initial signs of metabolic progression has eventually pseudoprogression. Importantly, survival analysis revealed a longer OS for patients with pseudoprogression compared to those with cPMD; this difference in survival may not be statistically significant, which is very likely attributed to the small number of patients in the pseudoprogression group, but a clear trend was recognized in the respective Kaplan–Meier curves. Secondly, we demonstrate that the incidence of pseudoprogression can be markedly decreased after application of novel response criteria and identification of signs of irAEs. Thirdly, patients eventually responding to ICIs with cPMD exhibit a higher SLR_mean_ after the first 2 cycles of treatment than those showing pseudoprogression.

The detection of non-responders already after administration of the first ICIs cycles carries significance, since it can limit the treatment-associated toxicity and financial burden in patients that are unlikely to profit from ICIs [36]. In our cohort, 83.9% of patients with early uPMD (according to EORTC) had a confirmed metabolic progression when scanned at a later time point. These patients would potentially benefit from an early cessation of the non-effective, potentially toxic treatment and a change in therapeutic management at the appropriate time.

On the other hand, the identification of uPMD at an early time point comes at a cost: the misdiagnosis due to the phenomenon of pseudoprogression of some late responders as PMD would deprive these patients of the beneficial effect of immunotherapy. Indeed, a non-negligible number of patients (16.1%) eventually showed a subsequent remission of the initial uPMD. This finding suggests that pseudoprogression is not uncommon, which is in line with the results of a recent study by Pires da Silva et al. In that study, involving 140 melanoma patients treated with combined immunotherapy, one-third of all patients with progressive disease -according to RECIST 1.1—had eventually pseudoprogression and exhibited similar survival compared with non-progressors [33].

These challenging results call for a more detailed analysis of the specific PET/CT findings of the 5 patients with pseudoprogression, in order to possibly identify imaging characteristics suggestive of this phenomenon and develop approaches that could address it. Specifically, in 2 patients of the pseudoprogression group—both of which showed a very good clinical response—misdiagnosis could be tackled after application of the recently proposed PERCIMT, instead of the EORTC criteria. The cornerstone of PERCIMT is the finding that the absolute number of newly emerged ^18^F-FDG-avid lesions is more predictive of clinical outcome than SUV changes during melanoma immunotherapy [17]. In particular, neither a mere increase (> 25%) in tumor SUV nor the development of one new hypermetabolic lesion in follow-up PET/CT scan mean disease progression per se, as defined by the EORTC criteria. Instead, PERCIMT suggest the application of a threshold of four newly emerged lesions—with a decreasing cutoff of lesion number as the functional diameter of the lesions increases—for patient classification to progressive disease (Table 1) [37]. The hitherto preliminary application of PERCIMT has shown promising results in patient stratification [21, 38–41].

Besides PERCIMT, various novel metabolic response criteria have been recently proposed as alternative approaches to the conventional PET criteria—mainly the PET Response Criteria in Solid Tumors (PERCIST)—which were based on data derived from cohorts under cytotoxic therapies [42]. These novel criteria seem to outperform PERCIST regarding their prognostic value in immunotherapy. In particular, the iPERCIST criteria use the “wait and see” approach, requiring a dual time-point evaluation for confirmation of the initial signs of PMD on PET/CT. iPERCIST introduce two new categories of response, derived from iRECIST: the unconfirmed progressive metabolic disease (UPMD), defined at 2 months after start of treatment (equivalent to 4 cycles of therapy), and the confirmed progressive metabolic disease (CPMD), requiring another evaluation 4 weeks after manifestation of UPMD [18]. Another set of promising, novel criteria developed for immunotherapy evaluation are the imPERCIST, which follow the changes between and after the end of ipilimumab administration of the peak SUV of ^18^F-FDG in up to 5 measurable tumor/target lesions corrected for lean body mass (SULpeak), as suggested by PERCIST. However, imPERCIST have two major differences in comparison with PERCIST: firstly, PMD is not defined by the appearance of new lesions but exclusively by the increase of SULpeak. Secondly, the selection of target lesions at follow-up scan(s) is based on the 5 hottest lesions among all lesions in each scan—including newly emerging lesions -, irrespective of the distribution of lesions at baseline PET/CT imaging [19]. Indicatively, the application of iPERCIST and imPERCIST in the herein presented group of 5 patients with pseudoprogression after the initial 2 ICIs cycles would have led to correct classification of one patient as SMD, thus, reducing the pseudoprogression cohort to 4 patients. Respectively, after the administration of 4 cycles of therapy, 2 more patients would show metabolic benefit to treatment (SMD) according to these criteria.

With further consideration to the pseudoprogression group, one patient developed a transient sarcoid-like lymphadenopathy of the mediastinal and hilar nodes. He eventually showed a very good clinical response. In clinical practice, the emergence of this response pattern during immunotherapy can create a diagnostic dilemma for imaging specialists and clinicians, since it may mimic disease progression. Based on the present findings and the steadily growing literature in the field [43, 44], sarcoid-like lymphadenopathy should be not be interpreted as disease progression, but rather acknowledged as a possible irAE, which may be, moreover, associated with improved tumor response and disease control [33, 45, 46]. Therefore, in such cases we recommend the performance of at least one follow-up PET/CT scan or histopathological examination of the respective imaging findings.

Finally, the remaining 2 cases were difficult to identify as potential pseudoprogression patterns. One patient showed a transient, generalized lymphadenopathy, which constitutes a rather sporadic response pattern under immunotherapy, previously described in some case reports [47]. The second patient demonstrated a marked advance in both the number and metabolism of the baseline lesions, which subsided after 6 months, representing an example of the more delayed but durable responses, sometimes observed under ICIs [48].

Aside from applying various response criteria, our analysis also involved the semi-quantitative assessment of immune activation signs on PET as a supplementary approach for prediction of the immunotherapy effect. This was attempted through the calculation of the ratio of ^18^F-FDG uptake (SUV) in the spleen and the liver (SLR). Our results showed that SLR_mean_ after the first 2 ICIs’ cycles was significantly higher for patients eventually showing cPMD compared to those with pseudoprogression, suggesting that increased spleen glucose metabolism under ICIs may correlate with negative clinical outcomes. This finding is consistent with recently published literature investigating spleen metabolism by means of ^18^F-FDG PET, which highlighted the significant association between a high SLR and a poor outcome to immunotherapy [23, 49]. At the same time, these results should be interpreted with caution, since other studies in the field—although not explicitly involving SLR calculations—have shown a less contributory role of spleen metabolism in differentiating responders from non-responders to ICIs [46, 50, 51], supporting the opinion that spleen metabolism is a “double-edged” biomarker with debatable results [52]. On the whole, the investigation by means of functional imaging not only of the tumor but also of the patient’s host immune system gradually gains significance as potential surrogate marker of treatment response. Future prospective studies should determine whether this approach could indeed serve as prognosticator of immunotherapy.

Taken together, our findings show that the majority of non-responders can be identified early during immunotherapy, which may carry serious clinical implications. At the same time, approximately 16% of patients with uPMD eventually responds to treatment with remission of signs of pseudoprogression. These results support the use of early PET/CT scanning during immunotherapy, i.e. after application of two cycles of ICIs, but at the same time call for careful handling of the findings of early metabolic progression. In this context, the performance of another follow-up PET/CT at a second time-point for confirmation of early PMD is recommended. Given that the median time required for remission of pseudoprogression was 2.3 months in our cohort, and the fact that a long delay could risk disease decompensation to the point of rendering the patient incapable of receiving salvage chemotherapy, the late follow-up PET/CT examination may be performed approximately 8–10 weeks after initial, early progression. Besides that, we suggest the application of some practical tools for tackling the phenomenon of pseudoprogression, such as the usage of novel metabolic response criteria as well as the identification of sarcoid-like lymphadenopathy as a possible irAE rather than as a manifestation of disease progression. Finally, our results support the supplementary role of SLR_mean_ as potential prognosticator in melanoma patients under immunotherapy.

Our study has some limitations. Firstly, this is a retrospective analysis of prospectively acquired data with a relatively limited number of patients due to the strict inclusion criteria applied. A validation of these findings in larger patient cohorts would be therefore required. Moreover, several patients were not further examined with PET/CT—in terms of the present analysis—after the first 4 cycles of ICIs, since they were either clinically characterized as progressors and subsequently changed/stopped therapy, or they soon thereafter died. Theoretically, a confirmation of the cPMD findings with another PET/CT scan later on (after the 4 cycles), may have revealed remission of signs of pseudoprogression in some of these patients. However, the clinical definition of progressive disease was always predominant, leading to respective, early management changes. Another limitation lies in the fact that most of the patients (84%) described in the present series were treated with ipilimumab monotherapy, which is no longer the standard of care in melanoma; instead, melanoma treatment nowadays involves anti-PD-1 monoclonal antibodies, applied both as single agents and in combination with ipilimumab. Further, the vast majority of the PET/CT-positive findings were not histopathologically confirmed, which is, however, not usually possible in the clinical setting. Finally, this study focused on two sets of criteria, one conventional (EORTC) and one novel (PERCIMT). The other recently proposed, immunotherapy-modified PET response criteria, such as iPERCIST and imPERCIST, were applied only in a subcohort of the studied patient population (pseudoprogression group). However, we are in the ongoing process of evaluating the performance of all available PET criteria—conventional and modified—in an extended patient cohort, including not only patients with PMD but all classes of response to ICIs; these evaluations will be the topic of a future work of our group.

## Conclusions

In an attempt to investigate the phenomenon of early metabolic disease progression, we serially monitored with ^18^F-FDG PET/CT a cohort of metastatic melanoma patients under ICIs. Approximately five out of six eventual non-responders could be identified with PET/CT already after administration of the first two ICIs cycles, highlighting the potential role of interim PET/CT in early recognition of patients that are unlikely to profit from ICIs. These patients would potentially benefit from an early cessation of the non-effective, potentially toxic treatment and a change in therapeutic management at the appropriate time. On the other hand, 16% of patients with initial signs of metabolic progression was later proven to have pseudoprogression. Therefore, the performance of another follow-up PET/CT approximately 8–10 weeks after initial metabolic progression, in order to tackle the phenomenon of pseudoprogression, is recommended. Moreover, the usage of novel interpretation criteria and the identification of special types of radiological irAEs can further aid in reliable immunotherapy response evaluation. Finally, the investigation of spleen glucose metabolism, as part of the generalized host immune system activation under ICIs, may offer further prognostic information in melanoma patients undergoing immunotherapy.

## Data Availability

The dataset used and/or analyzed during the current study is available from the corresponding authors on reasonable request.
